# A new task format for investigating information search and organization in multiattribute decisions

**DOI:** 10.3758/s13428-014-0482-y

**Published:** 2014-06-06

**Authors:** Florence Ettlin, Arndt Bröder, Mirka Henninger

**Affiliations:** 1 Experimental Psychology, School of Social Sciences, University of Mannheim, L13,15, 68131 Mannheim, Germany; 2 Experimental Psychology, School of Social Sciences, University of Mannheim, Schloss, EO 265, 68131 Mannheim, Germany

**Keywords:** Information search and organization, Multiattribute decisions, Process tracing, Decision making

## Abstract

In research on multiattribute decisions, information is typically preorganized in a well-structured manner (e.g., in attributes-by-options matrices). Participants can therefore conveniently identify the information needed for the decision strategy they are using. However, in everyday decision situations, we often face information that is not well-structured; that is, we not only have to search for, but we also need to organize the information. This latter aspect––subjective information organization––has so far largely been neglected in decision research. The few exceptions used crude experimental manipulations, and the assessment of subjective organization suffered from laborious methodology and a lack of objectiveness. We introduce a new task format to overcome these methodological issues, and we provide an organization index (OI) to assess subjective organization of information objectively and automatically. The OI makes it possible to assess information organization on the same scale as the strategy index (SI) typically used for assessing information search behavior. A simulation study shows that the OI has a similar distribution as the SI but that the two indices are a priori largely independent. In a validation experiment with instructed strategy use, we demonstrate the usefulness of the task to trace decision processes in multicue inference situations.

**Electronic supplementary material:**

The online version of this article (doi:10.3758/s13428-014-0482-y) contains supplementary material, which is available to authorized users.

Imagine that you are about to assemble a research team for your next project. From all the candidates who applied, you need to identify those who will be most suitable for your team. How do you proceed? Usually, you get information about the applicants such as CVs, letters of recommendation, and certificates. The information about the applicants’ skills can be used to infer how suitable the applicants are. This is a typical example of a multicue inference task: Cues (aspects of the CVs) provide information about different options (the job candidates), among which the decision maker needs to identify the best one(s). Further examples of multicue inference situations are diagnostic situations, in which the cues are symptoms and the options are different diseases or disorders. As a final example, information about companies may be used to infer which company’s shares to invest one’s money in. These kinds of tasks exist in the domain of preferences and of inferences. In the former domain, the information units are usually referred to as *attribute information,* and in the latter, they are referred to as *cue information*. Since we used an inference task in our experiment, we will generally use the term cue information unless we refer to previous research from the domain of preferences.

Different strategies and factors have been investigated to describe how the decision maker may use cue information and to identify influences on the selection of a decision strategy, respectively (Bröder, 2000b, 2003; Gigerenzer, Todd, & the ABC Research Group, 1999; Payne, Bettman, & Johnson, 1988, 1993; Svenson, 1979). The influence of time pressure (see, e.g., Payne et al., 1988; Rieskamp & Hoffrage, 1999; see Bröder, 2000b, for different results) and information costs (see, e.g., Bröder, 2000b, 2003; Newell & Shanks, 2003) on strategy selection are among the most investigated factors. The decision strategies investigated with multicue decisions differ in the amount of information considered as well as in additional aspects, which we will introduce below. These strategies are presumably more conveniently applied to well-structured than to unorganized information (see, e.g., Bettman, 1975, on processability). However, this step in the decision process––structuring information to increase processability––has so far not gotten much attention in research on decision strategies. One reason is probably that no standardized and easy-to-use research tools exist.

The goal of the new task presented here is to overcome this issue. We introduce a task format for the investigation of decision strategies in multicue decision tasks, a format that is based on and extends the commonly used Mouselab paradigm (Johnson, Payne, Schkade, & Bettman, 1989) but which is more flexible than the latter. In the Mouselab paradigm, decision-relevant information is hidden in boxes on the screen and can be acquired with the mouse device. This method allows tracking the information-acquisition process. In addition to the typically investigated variables choice, information search, and decision time, our task makes it possible to assess the subjective organization of information in a standardized manner. The new index we introduce quantifies subjective organization on the same scale as the *strategy index* (SI) that is often used for characterizing information search (Payne, 1976). Hence, we develop a paradigm in which search and organization can be investigated both simultaneously and independently from each other. Furthermore, the analysis of subjective organization is less laborious and more objective in comparison with previous approaches. With this task, we would like to shift attention to an aspect of the decision process that is oftentimes skipped, by providing participants with preorganized information. However, we argue that the subjective organization of information might provide valuable insights into the decision process in information-based decisions.

Before we present this new task format, we discuss the importance of spatial organization of information and continue with an overview of decision strategies for multicue decisions and of methods commonly used to investigate those. We then summarize previous research on information presentation format and on information organization. Thereafter, we introduce the new task for tracing information-organization behavior in multicue decisions and the organization index. With simulations, we show that important requirements for the new index are met. Next, we present a validation study with the new task and discuss the results, focusing on the importance of information organization for the application of decision strategies. Finally, we discuss limitations and possible future developments of the task, which make it possible to measure information organization with fewer restrictions, and might therefore provide further insight into how information is used in multicue decisions.

## Information organization, decision strategies, and previous research approaches

### Information organization

The way we organize information can be much more than just finding a place for things. According to Kirsh (1995) the way we organize items in space “is an integral part of the way we think, plan and behave” (p. 31). The idea behind this is that organization (in space) is used to reduce time and cognitive effort needed for a task. In addition, reorganizing information can help accentuate categories, and it facilitates visual search; that is, it gets easier to find information and to keep track of it (Kirsh, 1995).

Whenever we cannot freely organize the space around us, we might adapt by using different strategies. For instance, Ballard, Hayhoe, and Pelz (1995) showed that eye movements are adjusted and used to economically deal with tasks. The authors investigated eye movements in a hand–eye coordination task, in which a block configuration model had to be copied. In this task, participants seemed to follow an online information-acquisition strategy to save working memory costs (i.e., looking back to the required information right before the information is needed). However, when the costs for the online strategy were increased by shifting the separate sections in the task further apart, participants relied more on working memory, which was reflected in fewer eye movements back to the area containing the model information that had to be copied. Similarly, Russo (1977) showed that the spatial arrangement of information can facilitate its use. By making price information more processable, higher performance (amount of money saved) was achieved. Importantly, making the information content more comparable by using unit prices alone was not as effective as when these unit prices were spatially assembled in a list rather than being tagged to the supermarket shelves.

In sum, the studies by Ballard et al. (1995) and Russo (1977) highlight influences of spatial arrangements on strategy use and on performance. In addition, the considerations by Kirsh (1995) emphasize that we spatially arrange items in a meaningful way that relates to the task at hand. These results and considerations suggest that the spatial arrangement or organization of information might be highly relevant for information-based decisions. Particularly, the idea of organizing information in order to save working memory might be central for the application of decision strategies in multicue decisions.

### Decision strategies

Strategies for multicue inference tasks differ in terms of the amount and the sequence of information search as well as in their choice predictions. According to the fast and frugal heuristics framework (Gigerenzer et al., 1999), the decision maker selects a strategy that is adaptive in the given situation. The strategies are typically divided into two broad categories: noncompensatory and compensatory strategies (see, e.g., Payne et al., 1988; Svenson, 1979). With a noncompensatory strategy (e.g., the *Take the Best* [TTB] heuristic; Gigerenzer & Goldstein, 1996), the less important cues are ignored; the decision is based on the most important reason, and no tradeoffs are made. In other words, for assembling a research team, a team leader using TTB identifies the information or skill he deems most important to identify a good team member––for instance, experience with the research topic––and compares the applicants on that skill. Only if there is more than one remaining applicant who excels on the specific skill, will the team leader compare the remaining applicants on the second most important skill. This procedure is continued until a decision can be made. Compensatory strategies, however, integrate less important cues and trade them off against more important ones. So, if the project leader applies a compensatory strategy such as the *Weighted Additive Rule* (WADD; Payne et al., 1988) or the *Equal Weight Rule* (EQW; Dawes, 1979), he will integrate all available information about each applicant (by first weighting each piece of information by its importance, in the case of WADD) and will then choose the applicant with the highest sum.

#### Investigating decision strategies

Two different approaches are commonly used to investigate what type of strategy a decision maker applied: the outcome-based approach and process tracing (see, e.g., Bröder, 2000a; Svenson, 1979). The former approach focuses on the choices people make. With items for which different strategies predict different choices, the comparison of a person’s actual choices with the strategy predictions makes it possible to identify the strategy the given person most likely used (see, e.g., Bröder, 2002, 2010; Bröder & Schiffer, 2003; Lee & Cummins, 2004; see also Rieskamp & Hoffrage, 1999). The other approach, process tracing, focuses on information search. The strategies described above may not just differ in their pattern of predicted choices but they especially differ in their information search and stopping rule. Whereas TTB prescribes cue-wise search for information, the compensatory WADD and EQW are usually associated with more option-wise search patterns (see, e.g., Bettman & Zins, 1979; Payne et al., 1988), and the latter strategies use (almost) all available information, whereas TTB stops information search as soon as a discriminating cue is found. The information search process is typically investigated with the Mouselab paradigm (Johnson et al., 1989), with eyetracking (e.g., Franco-Watkins & Johnson, 2011; Lohse & Johnson, 1996), or with verbal protocols (see, e.g., Jarvenpaa, 1989, 1990; Payne, 1976; Stone & Schkade, 1991; see Schulte-Mecklenbeck, Kühberger, & Ranyard, 2011, for a recent overview on process tracing approaches).

**Fig. 1:**
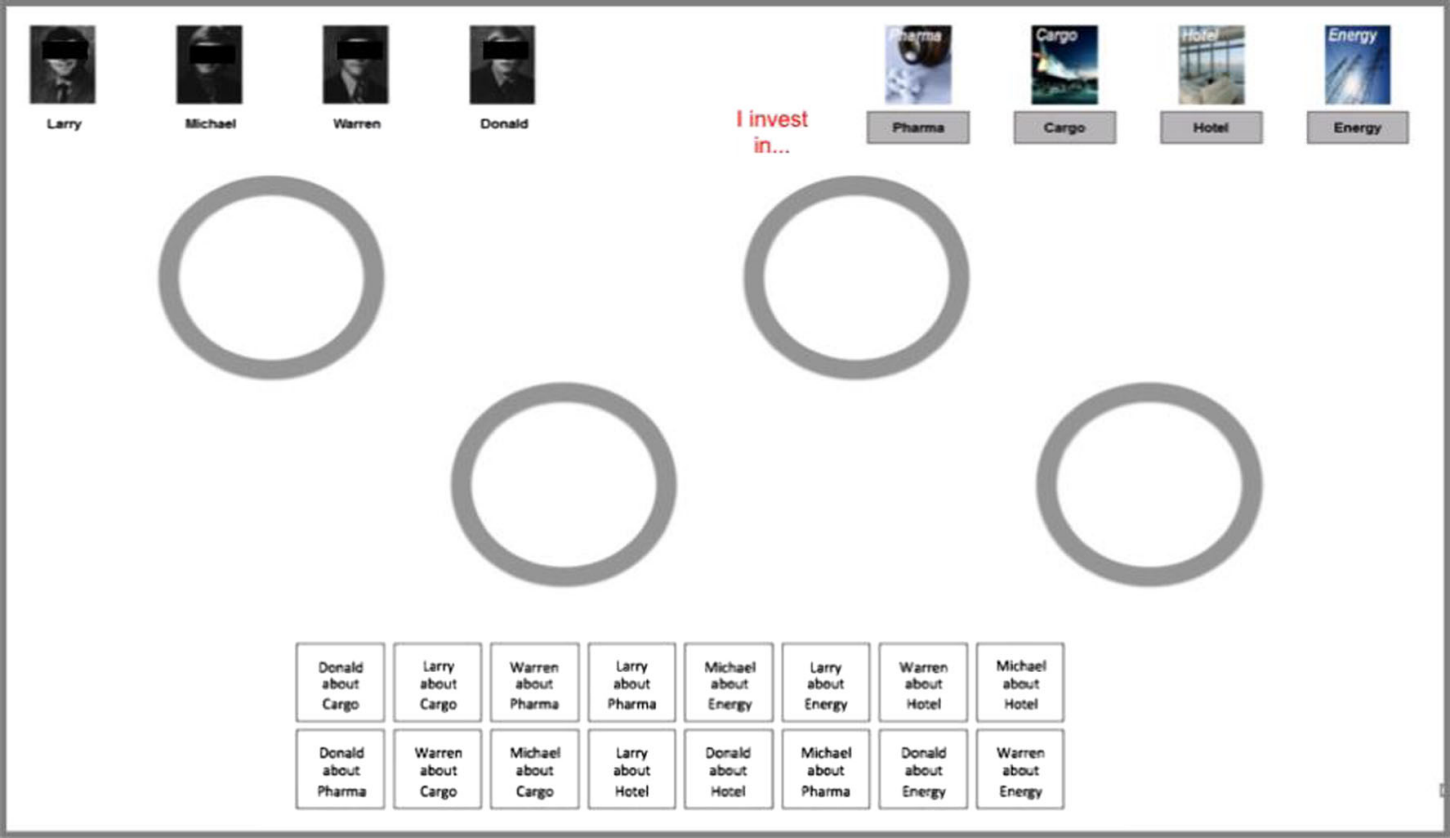
Example of the SOT: Details about the screen and the task are explained in the text

These approaches have the advantage of providing standardized methods for investigating decision strategies. However, this advantage comes with the cost of necessitating well-structured information presentation. Specifically, information is typically provided in a preorganized manner, for instance, in a cues-by-options matrix (for an example, see Fig. 1 in Bröder, Glöckner, Betsch, Link, & Ettlin, 2013). In the Mouselab paradigm, the information in the cells of the matrix is hidden, and participants need to click on the cells in order to acquire the information. Also, when eyetracking is applied, information is often provided in a matrix format––either with open information cells or with hidden information that is revealed as soon as the participant places a fixation on a cell (see Franco-Watkins & Johnson, 2011). The problem with this format is that the information search pattern applied to matrices may be influenced by the typical reading direction (Scherndl, Schulte-Mecklenbeck, & Kühberger, 2013). That is, when the attributes describing different choice options are presented in the rows of a matrix, search is more attribute-wise than when the attributes are presented in the columns. Furthermore, we rarely encounter information in matrices in everyday decision situations. The matrix format is rather an exception, which is used, for instance, in consumer reports. But other formats were also used in process tracing studies that applied methods such as eyetracking, flashlight, or mouse-response trajectories (see, e.g., Koop & Johnson, 2013; Schulte-Mecklenbeck, Murphy, & Hutzler, 2011; Visschers, Hess, & Siegrist, 2010). However, sometimes we even have to gather information from various different sources. Therefore, we do not only have to search for information, we also have to organize it by ourselves.

When introducing subjective information organization in multicue decision tasks, two main questions emerge. First, how does the organization of information influence the selection of decision strategies? And second, (how) do people organize information differently when they use different strategies with diverse information search and decision rules? In other words, is subjective organization of information used to reduce the costs of strategy application? Many years ago, decision scientists already addressed these issues (Coupey, 1994; Coupey & DeMoranville, 1996); however, research on the topic is still scarce, and previous approaches seem problematic. This is mainly because of crude manipulations, a lack of standardized methods to assess subjective information organization, and a lack of quantifiable measures of subjective organization.

## Previous approaches to investigating presentation format effects and subjective organization

The impact of information organization in multiattribute decisions has been investigated in two ways. The first approach confronted participants with different formats of preorganized information and assessed parameters of information search, decision quality, and confidence as the dependent variables. In this line of research, participants did not have the opportunity to organize the information by themselves. In the second approach, participants were given the opportunity to organize the given information, and this subjective organization was investigated both as a dependent variable and as a mediator to parameters of decision quality. We will characterize both approaches in turn.

### The influence of information presentation format on decision behavior

Bettman and Kakkar (1977) took a first step toward understanding effects of information presentation format on decision behavior. In a multiattribute preference task, the authors showed that participants who received information that was organized according to brands (alternatives) more often searched in an alternative-wise (option-wise) manner and that those who received the information ordered according to attributes searched instead in an attribute-wise (cue-wise) manner. So even though the same information was presented to all of the participants, their information acquisition patterns strongly differed depending on the presentation format.

This finding is not very surprising, however, given that the grouping of information in a brand- versus attribute-wise manner was manipulated using different booklets; that is, in the Brand Condition, there was a separate booklet for each brand containing all attribute information on a specific brand. In the Attribute Condition, the setup was analogous, with a separate booklet for each attribute. Therefore, acquiring information along the dimension according to which it was grouped (i.e., looking through one booklet after the next) was faster and more convenient than using a search strategy deviating from this pattern. Specifically, if a participant in the Brand Condition wanted to search for information in an attribute-wise manner, she had to switch booklets after each piece of information she gathered. Hence, the manipulation affected not only the saliency of the brand versus attribute dimensions, it also entailed high opportunity costs for applying a search strategy that mismatched the format.

Bettman and Zins (1979) continued this line of research by not only focusing on the presentation format but by considering the format *and* the task someone intended to apply. According to their task–format congruence hypothesis, for a given task and information format, “the degree of congruence between the processing characterizing the task and that encouraged by the format affects performance” (Bettman & Zins, 1979, p. 143; see also Vessey, 1991, on the idea of cognitive fit). The authors tested the congruency idea by providing specific strategy instructions (*tasks*), which differed with regard to whether option- or cue-wise processing was required, and by combining them with different *formats* (i.e., brand, attribute, and matrix format). The resulting conditions differed in their degree of congruence. According to the task–format congruence hypothesis, a task requiring brand-wise processing, for instance, should be easiest with the matrix, next easiest with the brand format, and hardest with the attribute format. In accordance with the hypothesis, participants adapted the decision time as a function of congruency (Experiments 1 and 2). Accuracy was not affected (Experiments 1 and 2), though. In addition, there was some support for the effect on subjective reactions (e.g., confidence, Experiment 2). But when participants had to choose a format for a specific strategy instruction, the majority preferred the matrix format, and there was no matching of format to the task (Experiments 1 and 2).

Again, when considering the formats provided, this finding is not surprising. The matrix was the only format with which all of the provided information was visible at once, on a single sheet of paper. The brand and attribute formats were such that each brand and each attribute, respectively, was described on a single sheet of paper on a tacked stack. But after eliminating the matrix format in a third experiment, the congruence hypothesis for format choice was not supported. In this final experiment, with one single decision trial, the results for decision time were not in line with the congruence hypothesis anymore, nor were the results for subjective reactions. To sum up, the support for the task–format congruence hypothesis was mixed, and as with Bettman and Kakkar’s (1977) experiment, the manipulation was rather crude and entailed opportunity costs for mismatching search strategies.

### Investigating subjective information organization

Coupey (1994) extended this work by giving people the opportunity to organize the provided information in a user-defined manner by simply providing participants with pen and paper. Participants’ notes were coded according to what kind of *restructuring* (i.e., changes to the information display) participants applied (i.e., whether they used editing, rearranging, etc.) and these coded notes provided the basis for the evaluation of the hypotheses. In the tradition of the cost–benefit idea (e.g., Beach & Mitchell, 1978; Payne et al., 1993), Coupey hypothesized that people would evaluate the costs and benefits of restructuring; that is, when restructuring is made easy by providing scratch paper, people will use the opportunity to restructure the display to facilitate the application of a more normative, alternative-based compensatory strategy. However, when the restructuring needs to be done in working memory, participants would rather rely on a simpler, noncompensatory strategy than restructure the information in their heads to be able to apply a compensatory strategy. This was indeed the case: Of the participants provided with scratch paper, 94 % used an alternative-based strategy, compared with only 40 % of those who were not allowed to take notes. In addition, the amount of restructuring depended on how well-structured the initial display was.

Coupey’s (1994; see also Coupey & DeMoranville, 1996) approach was certainly a step forward with respect to the investigation of information organization. However, we argue that apart from being extremely laborious, the method based on participants’ notes also entails a high degree of subjectivity because of the necessity of coding. A further line of research related to information organization is the investigation of information usage in quasinaturalistic risky choice decisions, with the active information search paradigm of Huber and colleagues (Huber, Wider, & Huber, 1997). Here, the focus is on what kind of information is asked for in a decision task when no information is provided in addition to the basic scenario. But as with most approaches, there is no separation of search sequence and information organization.

There is a general lack of standardized methods to elicit and a lack of measures to quantify subjective organization. However, we consider the organization of information an important aspect of the decision process that needs decision researchers’ attention. Especially nowadays, information is widely available and easily accessible for most of us. That is, whenever we want, or need, to make an information-based decision, we not only have to search for information, but we also have to filter and then organize it in order to be able to conveniently apply a decision rule. The new task we introduce was developed to make information organization assessable and analyzable in a more convenient and standardized manner.

## A new tool: The search and organization task (SOT)

Our new tool is based on the Mouselab paradigm, with the addition that not only information acquisition and choices but also subjective organization of information can be assessed and analyzed. We provide an index that quantifies information organization on the same scale as Payne’s (1976) index for information search, which is commonly used to assess information-acquisition patterns. Therefore, information organization becomes readily measurable.

With the SOT, a participant who starts working on a decision task sees a display that may look like the screenshot in Fig. 1. In the top left corner of the display, four cues are presented. In the example, these are four brokers who are ordered according to their importance, from left to right, with the leftmost broker being the one with the best predictions. In the top right corner, four choice options (here, market segments) are presented; their order of presentation is newly randomized for each decision trial. The 16 pieces of cue information, which result from fully crossing the four cues and the four options, are presented in the two rows of boxes in the lower half of the screen and are arranged in random order. As in the Mouselab paradigm, the information is hidden. The participants’ task is to identify the best market segment in several decision trials. In each trial, they start with a display that looks like the screen in Fig. 1. In order to make the inference decision, the participants can then acquire information from the brokers about the options by clicking on the piece of information (in the bottom rows) they are interested in. In this first step, the information label in the box that was clicked on disappears. In a next step, the participant needs to click into one of the four circles in the middle of the screen. Then the information label as well as the value of the information (“yes”/”no”) appears in that spot. With this basic version of the task, the only restriction is that a maximum number of four pieces of information fits into each of the circles. The participants are free to acquire information in any order they like and also to organize or group it in any way they desire (within the four circles). After each decision, the cue information for the next trial is hidden in a new random order in the two rows in the lower half of the screen. Again, participants are free to search and organize according to any preferred sequence and pattern, respectively.

In contrast to some Mouselab setups, once acquired, the information in the SOT remains visible for the whole duration of a trial. Because of this difference, reacquisitions cannot be analyzed with the SOT. However, investigating benefits and strategies of spatial layout is only useful for visible information. Eyetracking may be used to reveal how people make use of the information after it has been organized in the circles.

In addition to search order, amount of acquired information, choices, and decision times that are usually registered and analyzed in Mouselab or eyetracking studies, the SOT allows the assessment of subjective organization via the arrangement of information in the circles. That is, for each decision trial, the sequence in which the information is clicked on in the two bottom rows can be turned into Payne’s (1976) strategy index, SI. The SI is a measure of relative cue- versus option-wise search for information and is computed as the number of option-wise transitions minus the number of cue-wise transitions divided by the sum of the two numbers. It is measured on a scale from –1 (cue-wise search) to +1 (option-wise search). Our newly introduced organization index, OI, allows quantifying information organization on the same scale as the SI, ranging from –1 (cue-wise grouping) to +1 (option-wise grouping). The OI is computed as follows:

$$ OI=\frac{\left[\left[{\displaystyle {\sum}_{j=1}^4\left( \max \left( SameOptio{n}_{circle\kern0.5em j}\right)-1\right)}\right]-\left[{\displaystyle {\sum}_{j=1}^4\left( \max \left( SameCu{e}_{circle\kern0.5em j}\right)-1\right)}\right]\right]}{\left[\left[{\displaystyle {\sum}_{j=1}^4\left( \max \left( SameOptio{n}_{circle\kern0.5em j}\right)-1\right)}\right]+\left[{\displaystyle {\sum}_{j=1}^4\left( \max \left( SameCu{e}_{circle\kern0.5em j}\right)-1\right)}\right]\right]} $$

with max(*SameOption*_*circle j*_) as the maximum number of pieces of information describing the same option in circle j and with max(*SameCue*_*circle j*_) being the analogous value for cues. Subtracting 1 from each maximum count is necessary for scaling the index from –1 to +1. In the Appendix (see supplemental material), we provide R (R Core Team, 2013) code for the computation of the OI for one decision trial (code for a task with four options and four cues; examples of how to input data are provided with the code).

Hence, it is not necessary to code or classify messy notes on scratchpads, as in Coupey’s (1994) approach. Rather, information organization is directly assessed and automatically computed in each decision trial. Of course, our standardization comes with the limitation of restricting the organization to a cue-wise versus option-wise grouping. But we argue that these are probably the most relevant dimensions, and since the number of cases of the two dimensions is equal and is also equal to the number of circles, there is no a priori bias toward either of the two dimensions. In addition, we argue that the disadvantage of this limitation is outweighed by the advantage of standardization and objectivity.

### Flexibility of the SOT

The above-outlined explanation describes the basic version of the SOT to illustrate its logic. In fact, the task can be adjusted and modified in many ways. First, the *payoff environment* can be manipulated. This influences the adaptivity of the different decision strategies. Second, the *search environment* can be manipulated. Instead of leaving the sequence of information acquisition to the participant, one can predefine an acquisition sequence. For instance, with an option-wise restriction, participants are forced to acquire all information about one option after the next. Alternatively, search can be restricted accordingly in a cue-wise manner. Finally, the researcher can manipulate the *organization environment*. The organization environment may be changed by restricting the possibilities for the grouping of information (e.g., again in an option- or cue-wise manner) or by using a different display. Needless to say, the display depicted in Fig. 1 is only one example of an arbitrary arrangement of elements. For our initial studies, we used circles, to avoid any resemblance to the usual matrix format. However, as long as the basic idea of spatial grouping of elements is maintained, there is no restriction to the actual design of the display (see the General Discussion).

In summary, the SOT is flexible and allows for far subtler format manipulations than have been used in previous studies (see, e.g., Bettman & Kakkar, 1977; Bettman & Zins, 1979). Furthermore, the method is standardized and automatic, and does not need to be coded by the researcher. Finally, the OI is an objective measure for information organization that is comparable to the SI measure for assessing information search. In the remainder of the article, we will present simulations and a validation study that show that basic requirements for the OI are fulfilled and that people seem to be able to make use of the possibility to organize information in multicue inference tasks.

## The organization index: A simulation study

An important requirement for the OI is its a priori independence from the SI. In other words, certain sequences of search should not be associated statistically with specific patterns of subjective organization. Hence, artificial correlations should be ruled out. To investigate this issue, we conducted a simulation study, generating 100,000 random trials of information search and organization sampled from the population of all combinations possible with four cues and four options. The function *sample()* in R (R Core Team, 2013) was used to first determine the number of information pieces acquired by a simulated decisionmaker in a trial [*x* = sample(1:16,1)] and then to sample the *x* items from all information pieces [*y* = sample(1:16,*x*)].

**Fig. 2:**
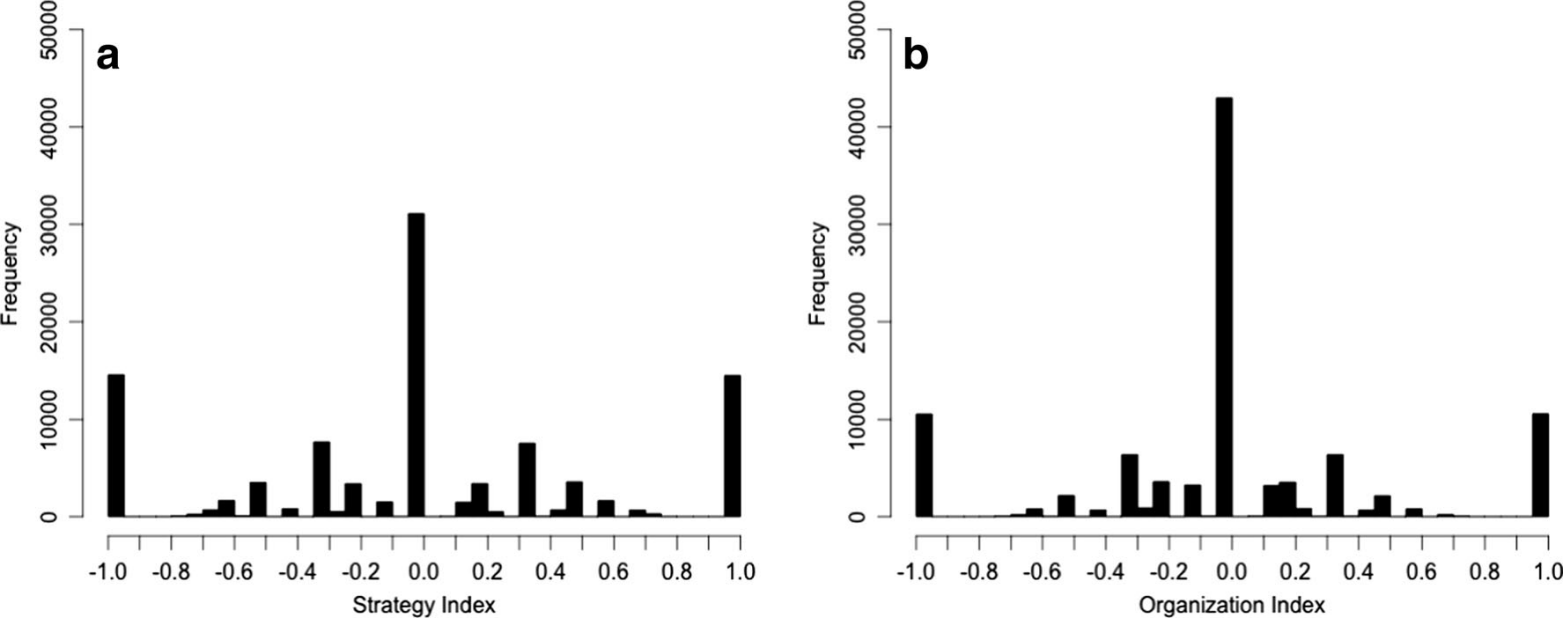
(A) Simulated SI. (B) Simulated OI

For each of these samples, the SI was computed. The resulting distribution of SIs is depicted in Fig. 2A. As expected for a representative random sample from the whole distribution of possible search patterns, the mean SI is zero; that is, neither cue- (negative numbers) nor option-wise (positive numbers; *M*_SI_ = 0.00, *Mdn*_SI_ = 0.00, 1st Quartile_SI_ = –0.33, 3rd Quartile_SI_ = 0.33). Importantly, the expected value of the SI is zero only in symmetric cases with the same number of options and cues (Böckenholt & Hynan, 1994). Although the *display* of information in our example is asymmetric (2 rows, 8 columns), the information *structure* is symmetric (4 by 4), and the latter aspect is relevant for the SI distribution.

In a second step, the random samples generated to compute the SI were randomly placed into *n* slots of the 16 slots representing the 16 positions in the four circles provided to organize information in each simulated trial. Again, we used *sample()* to randomly distribute the previously drawn samples to the 16 slots. From this, the OI was computed for each of the 100,000 samples. The resulting distribution of OIs can be seen in Fig. 2B. As with the SI, the mean OI is zero; that is, again random with respect to cue- (negative numbers) versus option-wise (positive numbers) grouping of information (*M*_OI_ = 0.00, *Mdn*_OI_ = 0.00, 1st Quartile_OI_ = –0.20, 3rd Quartile_OI_ = 0.20). In addition to being unbiased, both distributions show a similar shape, and both are symmetrical.

**Fig. 3:**
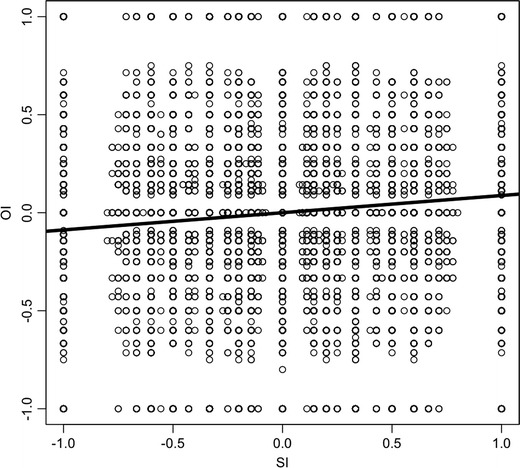
Scatterplot showing the relation of the SI and the OI (simulations)

As mentioned above, the two measures should not be a priori correlated. This requirement is largely fulfilled (*r* = 0.10, *R* ^*2*^ = 0.01, Kendall’s rank-order correlation coefficient τ = 0.06; see Fig. 3). This means, when we observe that participants’ information search and organization behavior is correlated, it will not be an artifact of the indices, but it will rather indicate a true behavioral matching of search and organization. So the SI does not restrict the possible OI values. But of course, there are certain cases for which the SI and OI are restricted. For instance, when only one piece of information is acquired, both indices need to be 0. Also, when only two pieces of the same cue (option) are acquired, for instance, then the SI is always –1 (1) and the OI can only assume the values 0 or –1 (1).

## Validation experiment

After checking the requirements for the OI, we conducted an empirical validation study of the SOT. We investigated information search and organization behavior with the instructed use of decision strategies. In one of the two between-subjects conditions, participants were instructed to use the compensatory EQW rule (*n* = 31); in the other condition, participants were instructed to use the noncompensatory TTB heuristic (*n* = 32). Hence, corresponding to the instructions for the decision strategies, we expect more option-wise search in the EQW Condition and more cue-wise search in the TTB Condition. If Bettman and Zins’ (1979) task–format congruence hypothesis (see also Vessey’s, 1991, cognitive fit hypothesis) holds, we also expect participants to organize the information in a way that best suits the strategy. Hence, in this validation study, we expect a high correspondence between SI and OI.

### Method

#### Participants

Sixty-three people [19 male; *M*_Age_ = 23 years (*SD*_Age_ = 5)] participated in the experiment in our laboratory at the University of Mannheim. The majority of the participants were students, with a few exceptions of employed people; the majority of the students were majoring in psychology. A chocolate bar was offered for participation. In addition, students could acquire course credits. The participants were randomly assigned to one of the two conditions (EQW or TTB Condition). Because of lower than chance level performance and because of a lack of compliance with respect to the use of the instructed strategy, we excluded 4 participants (see the Manipulation Check below). The remaining sample size allows for a statistical power > .90 to detect large effect sizes (*d* = .80, Cohen, 1988) in a *t*-test with α = .05.

#### Materials and procedure

Upon their arrival, participants were greeted, they signed a consent form informing them about their rights and duties as participants, and they were brought to one of six individual cubicles with a computer. After the experimenter had settled them and started the program, the participants worked through the decision task on their own.

For the validation experiment, the setup on the screen was basically identical to the one in Fig. 1, but the task content differed: The cues (top left) were four friends (Paul, Lars, Mike, & Jan) providing advice (in terms of “yes” and “no” hints) about the options (top right), namely, vehicles for traveling. The participants were put back in the year 1894 and were told that Phileas Fogg had just traveled around the world in 80 days. The participants’ task was to challenge Mr. Fogg and to travel around the world within half of that time. Therefore, they needed to identify the fastest vehicle (among sailing ship, steam train, carriage, and hot-air balloon) in each of 40 decision trials. Whenever they chose one of the slower vehicles for the upcoming route, they lost a day. The participants in the EQW Condition were told that their friends were all equally experienced and that it was best to choose the vehicle that was favored by a majority of their friends. In the TTB Condition, they were informed that their friends had different levels of experience with traveling around the world and that the friends were ordered accordingly, from left to right, with the most experienced friend being the one in the left corner. Participants were informed that they would achieve their goal by following the advice of the friend who was presented on the extreme left. Only if that friend could not decide between certain options, then the friend who was to the first friend’s right should be asked for advice on the options between which the first friend could not decide. The procedure should be continued in this manner when the second friend’s opinion still did not lead to a decision.

After the instructions about the task and about the nature of the SOT, the participants completed a practice trial before working through 40 decision trials. The 40 items were constructed such that 100 % accuracy could be achieved in each condition if the participants applied the instructed strategy. For half of the items, EQW and TTB made identical predictions, but the two strategies’ predictions differed for the other half of the items. The items were separated into four blocks, each containing an equal number of discriminating and nondiscriminating items (the separation into blocks was not obvious to participants). Within each of these blocks, the items were presented in random order. The pictures of the four options on the top right were randomized after each decision and so were the four cue patterns of each item. Therefore, there was no option or position of an option on the screen that was systematically preferred by either of the strategies. Depending on choice accuracy, the journey took 40 to 80 days.

After each decision, participants got feedback about the outcome of all four options and about the number of days their journey had taken so far. The feedback was binary: Each choice was associated with either one additional day of travel (winning option) or two additional days (the three losing options). For eight predefined items, participants were asked for a confidence rating after indicating their decision and before they got feedback. However, since this measure was assessed for piloting reasons for further studies and is not of relevance for the purpose of introducing the SOT, we will not take the confidence ratings into account in this article. After finishing the experiment, participants got the chance to provide verbal feedback to the experimenter about their experience, difficulties, or any other issues related to the task. Finally, those who were interested in the background and the goal of the experiment were informed about the details before they left the laboratory.

#### Hypotheses

We expected a negative SI in the TTB Condition and a rather positive SI in the EQW Condition. But since the strategy instruction implied the search rule (as well as the stopping and the decision rule) for both of the strategies, this is a manipulation check rather than a true hypothesis. That is, the TTB instruction clearly described a cue-wise search pattern. The instruction for EQW, however, described option-wise integration (decision rule) but did not explicitly prescribe any specific search pattern. With respect to the organization of information, we hypothesized that participants would group the information in a manner matching the search rule of the instructed strategy (i.e., both patterns were expected to be either more cue-wise or rather option-wise). This would also facilitate the application of the instructed decision rules in the respective conditions, because the information that is needed in close temporal proximity would be grouped in close spatial proximity.

### Results

#### Manipulation check

When the strategy instructions were followed, all 40 decisions could be made correctly, resulting in a score of 40 days. With four options per decision trial, chance level performance was at 25 % correct answers. Sixteen correct answers was the lowest score that was significantly different from chance (Binomial test, *p*_0_ = 0.25, *p*_success_ = 0.40, *p* = .042) and participants who achieved 15 or fewer correct answers (i.e., a score of [15*1 day + 25*2 days = ] 65 days or more) were therefore excluded. This was the case for 3 participants who all had been assigned to the TTB Condition.

In a next step, the adherence rates to both strategies were examined, and participants who made more choices in accordance with the TTB heuristic when they were in the EQW Condition, or vice versa, were excluded. This led to the exclusion of 1 more participant in the TTB Condition. Therefore, the final sample consisted of 31 participants in the EQW Condition and 28 participants in the TTB Condition whose choices were generally in line with the instructed strategy. In this sample, the overall mean score for number of days taken for the journey was 45.5 (*SD* = 7.3; *Mdn* = 42), which corresponds to a mean of 5.5 incorrect choices [13.8 %; *Mdn* = 2 incorrect choices (5 %)]. In the EQW Condition, the mean score was 45.5 days (*SD* = 7.9, *Mdn* = 41) and also in the TTB Condition, the mean score was 45.5 days (*SD* = 6.7, *Mdn* = 42).

Finally, the number of acquired pieces of information should be 16 in (almost) every decision trial for EQW. For TTB, however, the expected mean number of acquisitions for the 40 decisions was 8.5. Participants in the TTB Condition indeed acquired a mean of 8.6 pieces of information (*SD* = 2.5; *Mdn* = 8.6). In the EQW Condition, the mean number of acquired pieces of information was somewhat lower than expected (*M* = 10.9; *SD* = 3.9; *Mdn* = 12.0). Note, however, that certain cue constellations allow applying the EQW decision rule even if not all pieces of information have been uncovered (e.g., one need not uncover the last cue of an option with three negative cue values if there is a rival option with two already uncovered positive values).

#### Information search

**Fig. 4:**
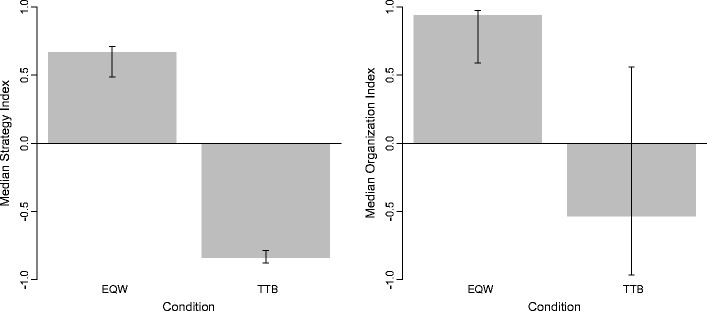
Median SI (left) and Median OI (right) for the EQW and TTB Conditions; error bars represent bootstrapped 95 % CI (2,000 samples)

As expected, the SI differed between the two conditions [Fig. 4 (left panel); *Mdn* = 0.07, Median test (for all Median tests, ties with the sample Median were put into the category of observations that were lower than the Median), χ^2^(1) = 37.63*, p* < .001, Cramér’s *V* = .799]. A nonparametric procedure was chosen because the distribution of the SI and OI in the groups was not normal or symmetrical. But the means (bootstrapped 95 % CI) showed the same pattern as the nonparametric results.

The information search pattern in the EQW Condition was in accordance with the expected option-wise search pattern: The Median SI in the EQW Condition was positive (0.67) and differed significantly from zero [one-sample Sign test (for all Sign tests, values equal to zero, i.e., equal to the Median tested under the null, were eliminated from the sample), η_0_ = 0, *n* = 31, *s* = 28, *p* < .001]. Also in the TTB Condition, the information search pattern was in line with the expected (and in this case specifically instructed) search rule for the strategy. The Median SI in the TTB Condition (–0.84) clearly indicated cue-wise search for information (one-sample Sign test, η_0_ = 0, *n* = 28, *s* = 2, *p* < .001).

#### Information organization

The OI also differed between the two conditions [Fig. 4 (right panel); *Mdn* = 0.59, Median test, χ^2^(1) = 6.17, *p* = .013, Cramér’s *V* = .323]. The information organization pattern in the EQW Condition was in accordance with the expected option-wise organization: The Median OI in the EQW Condition was positive and differed significantly from zero [one-sample Sign test, η_0_ = 0, *Mdn*_*n* = 26_ = 0.96 (*Mdn*_*n* = 31_ = 0.94; 5 participants’ OIs were zero), *s* = 23, *p* < .001]. In the TTB Condition, the OI showed the hypothesized negative sign indicating cue-wise grouping, but was not significantly different from zero [one-sample Sign test, η_0_ = 0, *Mdn*_*n* = 27_ = –0.70 (*Mdn*_*n* = 28_ = –0.54; 1 participant’s OI was zero), *s* = 11, *p* = .442].

As expected, the two process measures, SI and OI, were positively correlated in the sample, Kendall’s rank-order correlation coefficient τ = .44, *z* = 4.90, *p* < .001 (one-tailed). That is, participants searched for and organized information in a similar manner. This was true for EQW as well as for TTB users: Within each of the conditions, this positive relation was preserved [EQW Condition, Kendall’s rank-order correlation coefficient τ = 0.24, *z* = 1.88, *p* = .030 (one-tailed); TTB Condition, Kendall’s rank-order correlation coefficient τ = 0.24, *z* = 1.76, *p* = .039 (one-tailed)].

## General discussion

The following quote by Kirsh (1995) highlights an important aspect of information organization that we try to tap into with the SOT.

One of the most obvious and compelling ways of using space […] is to lay down items for assembly, in the order in which they are to be put together, touched, handed off, or otherwise used. Space naturally encodes linear orders, and higher orders if the agent has an encoding system in mind. The obvious virtue of encoding orderings in the world is to *offload memory*. (p. 51)

Information organization may tell us something about how information is used. The burden on working memory may be relieved, since the decision maker does not have to keep all the information in mind if he arranges information in a way that facilitates the application of a strategy.

Indeed, the validation experiment with instructed strategy use showed the expected difference in information organization behavior depending on the type of strategy (compensatory vs. noncompensatory). That is, participants organized the information according to how it was needed for the instructed decision strategy and such that it matched their information search behavior. Because of the grouping into categories, only the locations of the groups or chunks rather than the locations of up to 16 pieces of information need to be kept in mind (cf. Kirsh, 1995). Looking back and using the organized information should then become much more convenient than if the decision maker had to either gather the information from a random organization or if he just memorized it (cf. Ballard et al., 1995).

But for all that, the very act of organizing information is effortful and takes some time. So the question is whether the organization of information is as helpful as to justify the costs it entails (see Coupey, 1994, on the cost–benefit tradeoff for information restructuring). This seems to be the case for a compensatory strategy: The results for the OI were clear-cut in the EQW Condition, in which participants organized information matching the strategy (in Bettman and Zins’ [1979] terminology, they created a congruent situation). But in the TTB Condition, even though people searched for and organized information similarly, the pattern of organization was not as clear-cut as expected. This result suggests that the benefit of information organization is not equal for all strategies, and benefits of organizing information may be small for strategies that are very frugal.

The compensatory strategies integrate information, and it seems plausible that visual grouping of information helps to reduce the cognitive costs of integration and hence of applying a compensatory strategy. Noncompensatory strategies such as TTB do not integrate any information. In the case of four options, the four values of the most important cue need to be compared, for a start. As soon as the decision maker is clear on the next step (decision vs. which information to search for from the second-most-valid cue), the values of the first cue are not needed anymore. However, for TTB the sequence of information search should be highly relevant because of its clearly defined search rule. It will therefore be interesting to investigate whether, with the SOT, the search patterns are in accordance with the strategy models when the strategies are not instructed, a finding that was indeed observed with the Mouselab paradigm (see, e.g., Bröder & Schiffer, 2003). In addition, one could hypothesize that for TTB users, organization could become more relevant if they were restricted in their information search in a manner that is not favorable to the application of TTB.

To get back to the introductory example, if you as a team leader needed to decide which applicants to employ, you might actively search for information in addition to what you got from the applicants themselves. But a lot of information will already have been provided by the applicants, and it will therefore be ordered in an option-wise (or applicant-wise) manner. That is, for instance, in a job interview situation, you encounter one candidate after the next. However, if you intend to apply a noncompensatory strategy, you might just jot down the relevant information in order to be able to compare it for the different job candidates. In other words, information organization may be a relevant part of the decision process––especially when the decision maker has no or little control over the sequence in which she gets access to the information she is interested in. With the SOT, we provide a tool to investigate this aspect of the decision process.

### Comparison with previous research

Our results are in accordance with the task–format congruence hypothesis (Bettman & Zins, 1979; see also Vessey, 1991). We observed that participants matched their information organization behavior to the instructed strategy. Bettman and Zins (1979) did not observe this kind of matching in their studies when participants were allowed to choose a format for a given task. However, as mentioned previously, their format manipulation was rather crude. In contrast to the screen we provided, their brand-wise and attribute-wise formats did not allow participants to see all information at once. When the only format that actually displayed all the information on a single sheet (i.e., the matrix format) was taken out of the choice set, they still did not observe matching. However, they used only very few trials (i.e., a single decision in Experiment 3). In our experiment, participants went through 40 decisions and had time to develop their organization behavior and to routinize the decision process.

Finally, in comparison with Coupey’s (1994) research, there is one crucial difference between her and our conclusions worth highlighting: Whereas Coupey argued that the possibility to take notes and restructure information promotes the use of compensatory strategies, we concluded that people adapt their organization behavior to their strategy. The next step would be to test this with self-selected strategy use; that is, to investigate whether people still learn an adaptive strategy and organize the information accordingly or whether they jump to compensatory strategies.

### SOT: Limitations and outlook

Providing four circles for information organization may be viewed as a restriction of the method proposed here. We argue that this restriction is outweighed by the benefit of not having to code and categorize participants’ notes, which makes the SOT more standardized and objective than previous approaches. However, the circles prestructure the space for information organization, and differences in the kind of structuring applied to information have been shown to have an impact on decision behavior. For instance, different kinds of structuring have an impact on the pattern of information acquisition in risky choice (for discussions, see Brandstätter & Gussmack, 2013; Pachur, Hertwig, Gigerenzer, & Brandstätter, 2013; see also the section on “The influence of information presentation format on decision behavior” in this article). Note that in the literature just mentioned, the effect of structuring is reflected in information-acquisition behavior (i.e., in search patterns). The prestructuring in the SOT, however, concerns information organization *after* acquisition. Although we argue that the prestructuring in circles does not introduce any bias toward either options or cues, it may nevertheless introduce a demand effect toward grouping the information according to one of these two dimensions rather than in any other way. If the research focus is on cues and options and the comparison of organization and search, we argue that the prestructuring is appropriate and may yield less noisy data.

However, a future plan is to develop a version without circles or any other kind of structure. In this general version, people will be free to organize information on the screen in any way they like. Instead of the OI that we used in the prestructured version of the SOT, some distance-based index (DI) could be computed in this general version of the SOT. The DI could be based on the Euclidean distances between pieces of information. This general version of the SOT will reveal more about the importance of options and cues for participants’ subjective organization and about the question of how space is used when no structures are provided to suggest organizational patterns. However, the present version of the SOT is the first step in validating this new standardized method for the assessment of subjective organization. It keeps the strong focus on cues and options that has been present in previous research. The results of the validation study reveal that the OI captures an aspect of the decision process that differs from information search (as captured in the SI).

Problems may arise when the number of cues and options is increased. Given that the researcher wants to provide enough space for all of the information to be acquired and organized, the number of circles has to be identical to the number of options and cues. The number of options and cues should in turn be the same, in order to preserve symmetry and to not introduce a bias toward either of the dimensions. A to-be-developed version of the SOT without circles would provide more flexibility for this kind of variation and less demand effects.

Furthermore, the way the hidden information is provided can be varied. We chose labels with the structure “Cue X about Option Y” (e.g., “Mike about Carriage”) for the information boxes. One could argue that this order (cue first, option second) influences people’s information search and organization behavior. In the present study, we chose this labeling because it seemed natural with the content we used in the task. For future versions, different setups would be possible—for instance, allowing participants to collect the desired information by directly clicking the option name and the cue name in the display, in any order.

## Conclusion

We do not always get information in a well-structured manner, and sometimes we cannot get it in the preferred sequence. These are aspects of a decision situation that so far have not gotten the attention they deserve. As, for example, research by Bettman and Kakkar (1977), Bettman and Zins (1979), and Coupey (1994; Coupey & DeMoranville, 1996) shows, the effect of information organization has not been completely neglected. Nevertheless, the studies in this area have been surprisingly rare and have suffered from a cumbersome methodology.

With the SOT, we provide a useful new research tool. The new task does not require coding and categorization of notes. It retains the advantages of the Mouselab paradigm and adds to those the possibility of investigating subjective information organization. The OI provides an objective measure for information organization, which is computed online during the experiment. Our simulations, as well as the validation study, revealed promising results. The OI fulfills the necessary requirements as an objective quantification of information organization, which is comparable to the widely used SI measure for information search. And at least with the instructed use of strategies, the OI reveals the expected differences in information organization between users of a compensatory and users of a noncompensatory strategy. To conclude, the new task can be flexibly adapted to tackle various research questions concerning information organization, and we hope that decision researchers find it useful for doing so.

## Electronic supplementary material

Below is the link to the electronic supplementary material.

ESM 1 (DOC 33 kb)

## References

[CR1] Ballard DH, Hayhoe MM, Pelz JB (1995). Memory representations in natural tasks. Journal of Cognitive Neuroscience.

[CR2] Beach LR, Mitchell TR (1978). A contingency model for the selection of decision strategies. Academy of Management Review.

[CR3] Bettman JR (1975). Issues in designing consumer information environments. Journal of Consumer Research.

[CR4] Bettman JR, Kakkar P (1977). Effects of information presentation format on consumer information acquisition strategies. Journal of Consumer Research.

[CR5] Bettman JR, Zins MA (1979). Information format and choice task effects in decision making. Journal of Consumer Research.

[CR6] Böckenholt U, Hynan LS (1994). Caveats on a process-tracing measure and a remedy. Journal of Behavioral Decision Making.

[CR7] Brandstätter E, Gussmack M (2013). The cognitive processes underlying risky choice. Journal of Behavioral Decision Making.

[CR8] Bröder A (2000). A methodological comment on behavioral decision research. Psychologische Beiträge.

[CR9] Bröder A (2000). Assessing the empirical validity of the “Take-The-Best” heuristic as a model of human probabilistic inference. Journal of Experimental Psychology: Learning, Memory, and Cognition.

[CR10] Bröder A (2002). Take the Best, Dawes’ Rule, and compensatory decision strategies: A regression-based classification method. Quality and Quantity.

[CR11] Bröder A (2003). Decision making with the “adaptive toolbox”: Influence of environmental structure, intelligence, and working memory load. Journal of Experimental Psychology: Learning, Memory, and Cognition.

[CR12] Bröder A, Glöckner A, Witteman C (2010). Outcome-based strategy classification. Foundations for tracing intuition. Challenges and methods.

[CR13] Bröder A, Glöckner A, Betsch T, Link D, Ettlin F (2013). Do people learn option or strategy routines in multi-attribute decisions? The answer depends on subtle factors. Acta Psychologica.

[CR14] Bröder A, Schiffer S (2003). Bayesian strategy assessment in multi-attribute decision making. Journal of Behavioral Decision Making.

[CR15] Cohen J (1988). Statistical power analysis for the behavioral sciences.

[CR16] Coupey E (1994). Restructuring: Constructive processing of information displays in consumer choice. Journal of Consumer Research.

[CR17] Coupey E, DeMoranville CW (1996). Information processability and restructuring: Consumer strategies for managing difficult decisions. Advances in Consumer Research.

[CR18] Dawes RM (1979). The robust beauty of improper linear models in decision making. American Psychologist.

[CR19] Franco-Watkins AM, Johnson JG (2011). Decision moving window: Using interactive eye tracking to examine decision processes. Behavior Research Methods.

[CR20] Gigerenzer G, Goldstein DG (1996). Reasoning the fast and frugal way: Models of bounded rationality. Psychological Review.

[CR21] Gigerenzer G, Todd PM, the ABC Research Group (1999). Simple heuristics that make us smart.

[CR22] Huber O, Wider R, Huber OW (1997). Active information search and complete information presentation in naturalistic risky decision tasks. Acta Psychologica.

[CR23] Jarvenpaa SL (1989). The effect of task demands and graphical format on information processing strategies. Management Science.

[CR24] Jarvenpaa SL (1990). Graphic displays in decision making: The visual salience effect. Journal of Behavioral Decision Making.

[CR25] Johnson, E. J., Payne, J. W., Schkade, D. A., & Bettman, J. R. (1989). *Monitoring information processing and decisions: The mouselab system* (No. TR-89-4-ONR). Duke University Durham, Center for Decision Studies.

[CR26] Kirsh D (1995). The intelligent use of space. Artificial Intelligence.

[CR27] Koop GJ, Johnson JG (2013). The response dynamics of preferential choice. Cognitive Psychology.

[CR28] Lee MD, Cummins TDR (2004). Evidence accumulation in decision making: Unifying the “take the best” and the “rational” models. Psychonomic Bulletin & Review.

[CR29] Lohse GL, Johnson EJ (1996). A comparison of two process tracing methods for choice tasks. Organizational Behavior and Human Decision Processes.

[CR30] Newell BR, Shanks DR (2003). Take the best or look at the rest? Factors influencing “one-reason” decision making. Journal of Experimental Psychology: Learning, Memory, and Cognition.

[CR31] Pachur T, Hertwig R, Gigerenzer G, Brandstätter E (2013). Testing process predictions of models of risky choice: A quantitative model comparison approach. Frontiers in Psychology.

[CR32] Payne JW (1976). Task complexity and contingent processing in decision making: An information search and protocol analysis. Organizational Behavior and Human Performance.

[CR33] Payne JW, Bettman JR, Johnson EJ (1988). Adaptive strategy selection in decision making. Journal of Experimental Psychology: Learning, Memory, and Cognition.

[CR34] Payne JW, Bettman JR, Johnson EJ (1993). The adaptive decision maker.

[CR35] R Core Team (2013). R: A language and environment for statistical computing.

[CR36] Rieskamp J, Hoffrage U, Gigerenzer G, Todd PM, the ABC Research Group (1999). When do people use simple heuristics, and how can we tell?. Simple heuristics that make us smart.

[CR37] Russo JE (1977). The value of unit price information. Journal of Marketing Research.

[CR38] Scherndl, T., Schulte-Mecklenbeck, M., & Kühberger, A. (2013). *Neglected but pervasive: Further evidence for the influence of display orientation on information search*. Paper presented at the 6th JDM Workshop for Young Researchers, Berlin, Germany. Abstract retrieved from http://www.mpib-berlin.mpg.de/de/forschung/adaptive-rationalitaet/jdm-workshop

[CR39] Schulte-Mecklenbeck M, Kühberger A, Ranyard R (2011). A handbook of process tracing methods for decision research: A critical review and user’s guide.

[CR40] Schulte-Mecklenbeck M, Murphy RO, Hutzler F (2011). Flashlight–Recording information acquisition online. Computers in Human Behavior.

[CR41] Stone DN, Schkade DA (1991). Numeric and linguistic information representation in multiattribute choice. Organizational Behavior and Human Decision Processes.

[CR42] Svenson O (1979). Process descriptions of decision making. Organizational Behavior and Human Performance.

[CR43] Vessey I (1991). Cognitive fit: A theory-based analysis of the graphs versus tables literature. Decision Sciences.

[CR44] Visschers VHM, Hess R, Siegrist M (2010). Health motivation and product design determine consumers’ visual attention to nutrition information on food products. Public Health Nutrition.

