# Supplementary material for: A new task format for investigating information search and organization in multiattribute decisions
Source: Behav Res Methods. 2014 Jun 6;47(2):506–18. doi: 10.3758/s13428-014-0482-y (PMC4427654; doi:10.3758/s13428-014-0482-y)
Supplement: Supplementary file 1 — (DOC 33 kb) [file 13428_2014_482_MOESM1_ESM.doc]

**Appendix**

**R Code for the OI**

############ Explanations: Input for computation of OI ############################

#

# Input: a vector with 16 positions containing the organized information.

#

# Explanation: vector positions 1 to 4 represent the slots 1 to 4 in circle 1;

# vector positions 5 to 8 represent the slots 1 to 4 in circle 2;

# vector positions 9 to 12 represent the slots 1 to 4 in circle 3;

# vector positions 13 to 16 represent the slots 1 to 4 in circle 4;

# The numbers in the vector represent the kind of cue-information:

# Option1Cue1 = 1

# Option1Cue2 = 2

# Option1Cue3 = 3

# Option1Cue4 = 4

# Option2Cue1 = 5

# Option2Cue2 = 6

# Option2Cue3 = 7

# Option2Cue4 = 8

# Option3Cue1 = 9

# Option3Cue2 = 10

# Option3Cue3 = 11

# Option3Cue4 = 12

# Option4Cue1 = 13

# Option4Cue2 = 14

# Option4Cue3 = 15

# Option4Cue4 = 16

#example 1: completely option-wise grouping of information: Information in Circles

information_distribution <- c(1, 2, 3, 4, 5, 6, 7, 8, 9, 10, 11, 12, 13, 14, 15, 16)

#example 2: completely cue-wise grouping of information:

information_distribution <- c(1, 5, 9, 13, 2, 6, 10, 14, 3, 7, 11, 15, 4, 8, 12, 16)

#example 3: If a person does not acquire and organize all 16 pieces of information, use NA:

information_distribution <- c(1, 5, 9, 13, 2, NA, 10, NA, 3, NA, 11, NA, NA, NA, NA, NA)

############ Function computing the Organization Index ###########################

Organization_Index <- function(information_distribution) {

cues_per_circle <- rep(0, 16)

options_per_circle <- rep(0, 16)

# Loop over all four circles

for (circle in 1:4) {

# Loop over the slots in each circle

for (slot in 1:4) {

# This denotes the position of the data

# in the cues_/options_per_circle vector

slot_position <- (circle - 1)*4 + slot

slot_information <- information_distribution[slot_position]

# Find out which cue and option the information

# came from

cue <- (slot_information - 1) %% 4 + 1

option <- ceiling(slot_information / 4)

# Finally, increment the associated cell in

# the cues_/options_per_circle vectors

cues_per_circle[(circle - 1)*4 + cue] = cues_per_circle[(circle - 1)*4 + cue] + 1

options_per_circle[(circle - 1)*4 + option] = options_per_circle[(circle - 1)*4 + option] + 1

}

}

#subtract 1 from each sum of same cues and options per circle in order to scale the index from -1 to +1

#(single cues and options per circle equal 0, twice the same option or cue per circle equals 1 etc.)

cues_per_circle <- ifelse(cues_per_circle != 0, cues_per_circle - 1, cues_per_circle)

options_per_circle <- ifelse(options_per_circle != 0, options_per_circle - 1, options_per_circle)

#compute the OI:

# As a first step, transform the vectors into matrices,

# to make them easier to handle. This way, each matrix

# row corresponds to a circle, and each column to a

# cue (cues_per_circle) or option (options_per_circle)

cues_per_circle <- matrix(cues_per_circle, nrow=4, byrow=TRUE)

options_per_circle <- matrix(options_per_circle, nrow=4, byrow=TRUE)

# For each circle, we compute which cue or option was

# most present in this particular circle,

# and sum up these values over all circles

cues_in_circles <- sum(apply(cues_per_circle, 1, max))

options_in_circles <- sum(apply(options_per_circle, 1, max))

# From this, we can compute the organization index:

if(cues_in_circles + options_in_circles == 0){

OI = 0

} else {

OI = ((options_in_circles - cues_in_circles) / (options_in_circles + cues_in_circles))

}

return(OI)

}

Organization_Index(information_distribution)
